# Supplementary figures and images for: Sympathetic motor neuron dysfunction is a missing link in age-associated sympathetic overactivity
Source: eLife. 2024 Dec 3;12:RP91663. doi: 10.7554/eLife.91663 (PMC11614386; doi:10.7554/eLife.91663)

**Figure Source Data 1.** Original membranes corresponding to Figure 6 panel F.

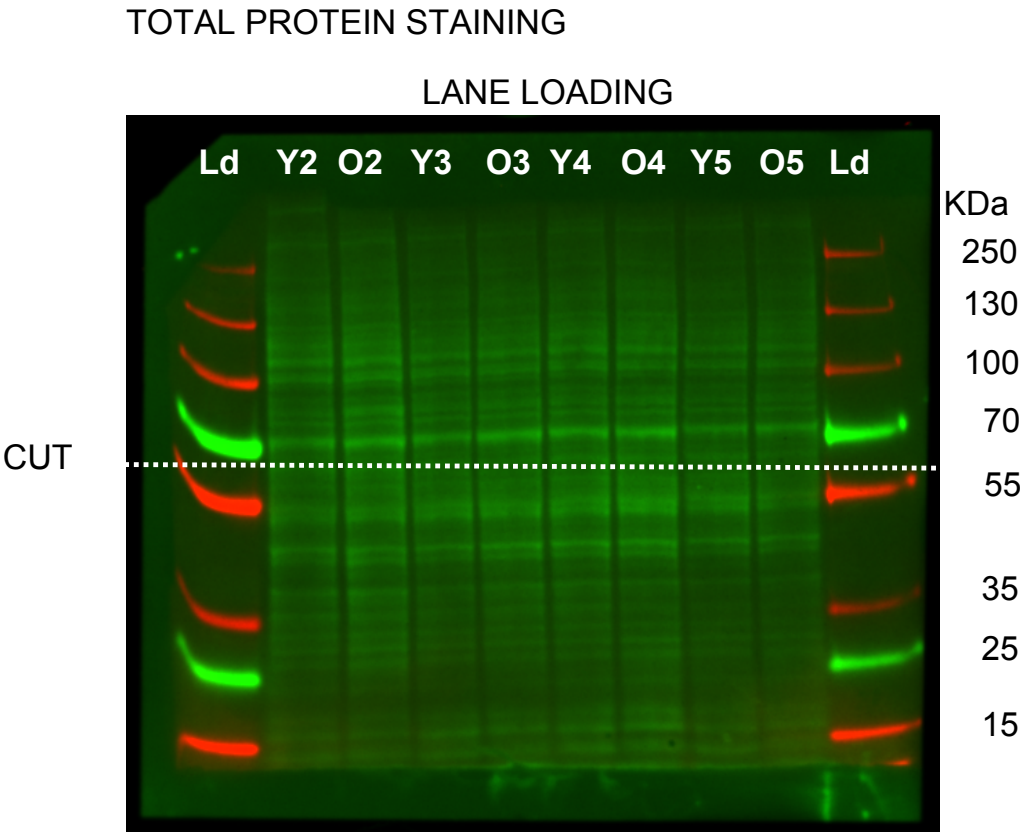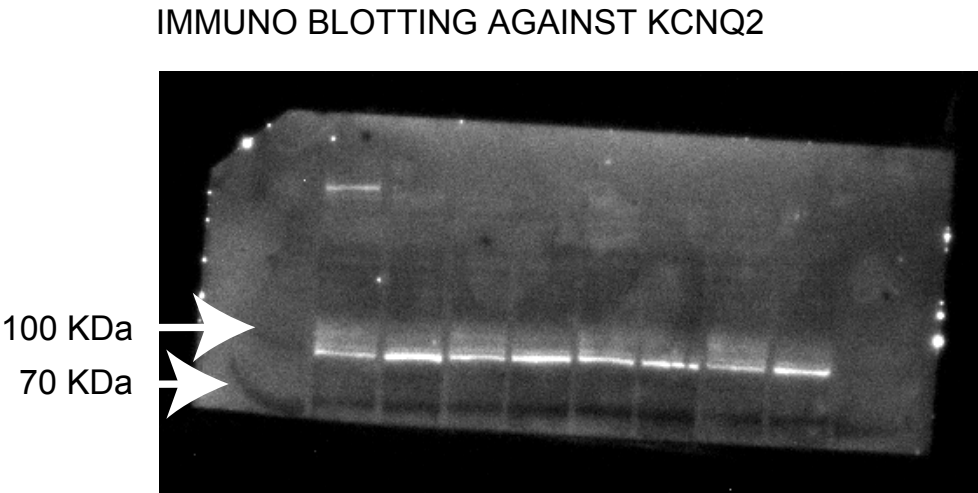

Supplement: Figure 6—source data 1. [file elife-91663-fig6-data1.pdf]

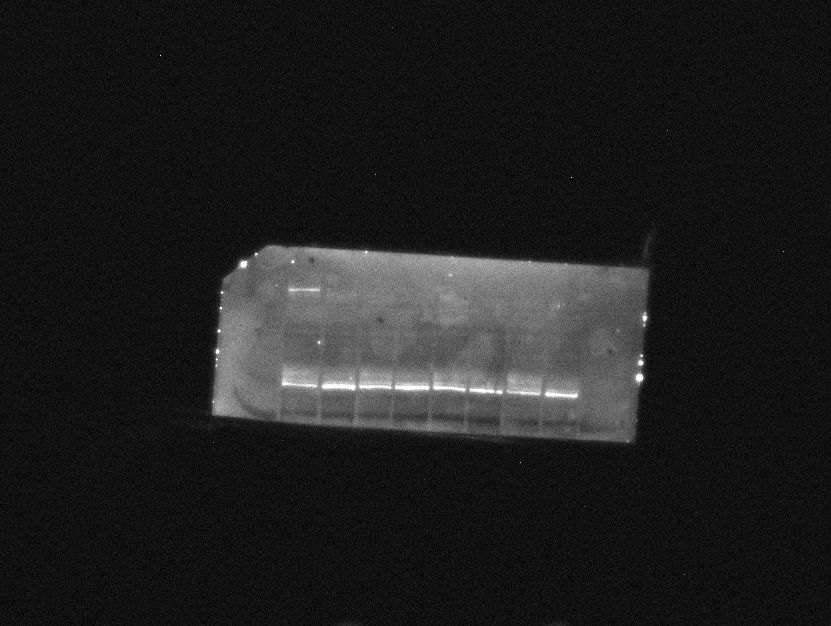

Supplement: Figure 6—source data 2. [file elife-91663-fig6-data2.zip › FIGURE 6 WB SOURCE DATA 2/RAW GELS/KCNQ2 HRP 16bit.png]

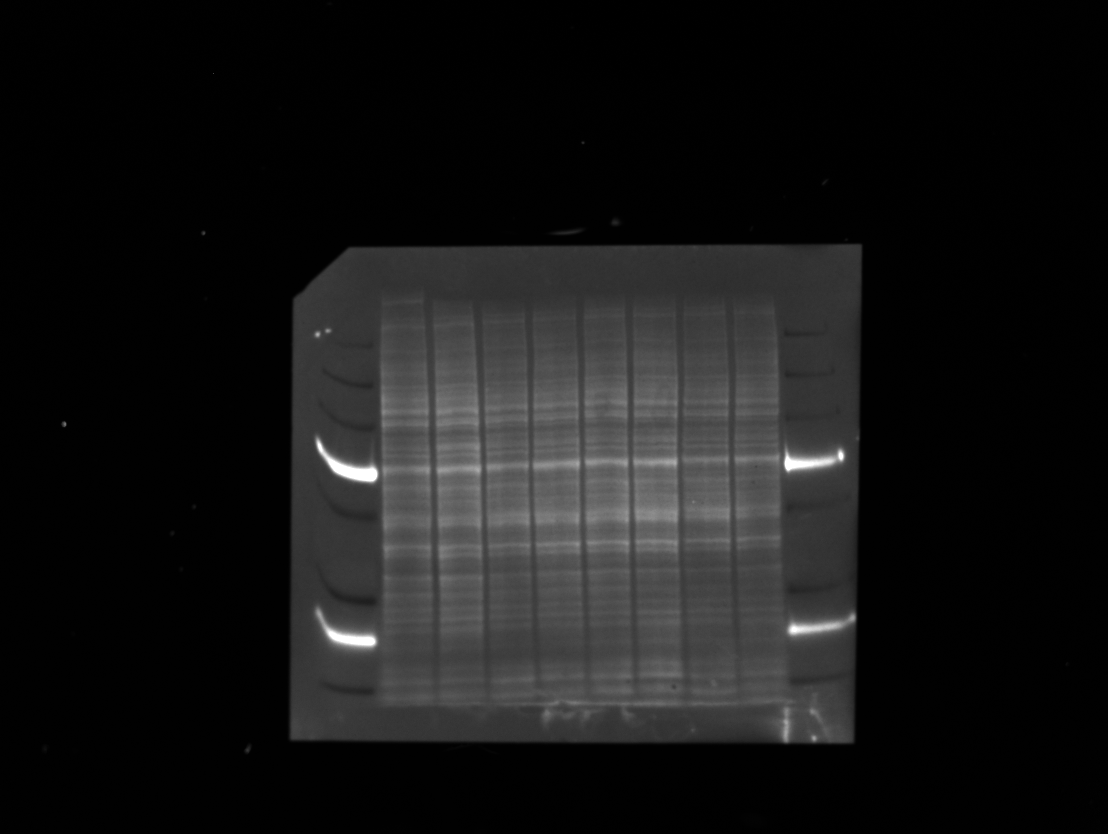

Supplement: Figure 6—source data 2. [file elife-91663-fig6-data2.zip › FIGURE 6 WB SOURCE DATA 2/RAW GELS/TOTAL PROTEIN_16bit.png]

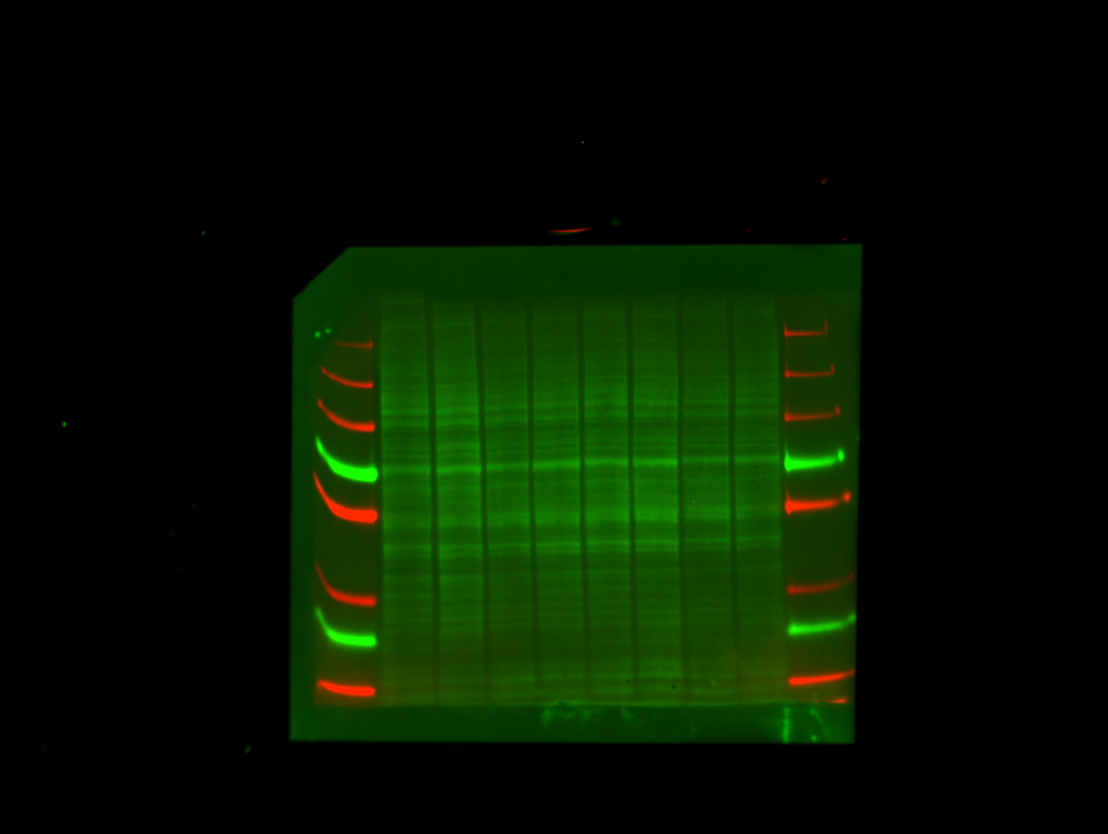

Supplement: Figure 6—source data 2. [file elife-91663-fig6-data2.zip › FIGURE 6 WB SOURCE DATA 2/RAW GELS/TOTAL PROTEINB_8bit.png]
